# Transverse carpal ligament tear at the trapezial ridge without fracture - the “trapezial ridge line” sign: two case reports

**DOI:** 10.1259/bjrcr.20220041

**Published:** 2023-01-23

**Authors:** Saroj K Golay, Ramy Mansour, Nicholas Riley, Yaron Berkowitz

**Affiliations:** 1 Department of Musculoskeletal Radiology, Nuffield Orthopaedic Centre, Oxford University Hospitals NHS Foundation Trust, Oxford, United Kingdom; 2 Nuffield Department of Orthopaedics, Rheumatology and Musculoskeletal Sciences, Botnar Research Centre, University of Oxford, Oxford, United Kingdom

## Abstract

A tear of the transverse carpal ligament attachment at the trapezial ridge without associated fracture has not been previously described. We present a detailed description of a 16-year-old Caucasian male patient treated at our institution, and a second supporting case of a 15-year-old Caucasian male patient with a similar mechanism of injury and diagnostic findings.

It is important to be aware of this ligament tear, as it may affect clinical management, is occult on computed tomography, and only detectable on magnetic resonance imaging, stressing the worth of magnetic resonance imaging in the setting of acute wrist trauma.

## Background

**Figure 1.**
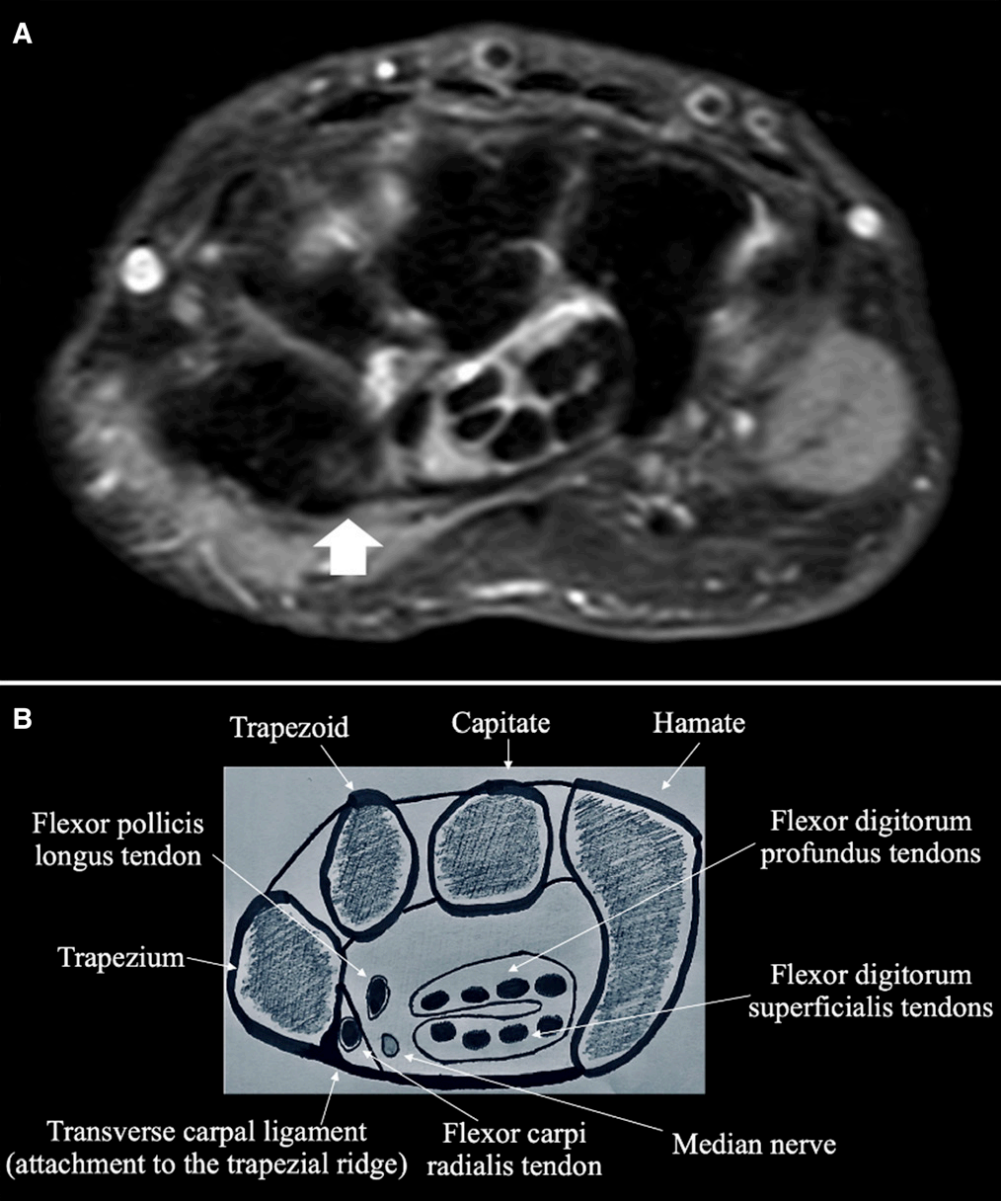
(**a**) Axial proton density fat supressed MRI image of the right hand of a control case patient demonstrating normal signal intensity of the intact transverse carpal ligament attachment to the trapezial ridge (arrow). (**b**). A representative anatomical diagram at the same axial level, courtesy of the authors, demonstrating the normal intact transverse carpal ligament attachment to the trapezial ridge and surrounding anatomical landmarks.

The transverse carpal ligament (also known as the flexor or transverse flexor retinaculum of the wrist) forms the roof of the carpal tunnel in the palm of the hand, attaching to the hamate and pisiform on the medial aspect, and the trapezium and scaphoid on the lateral aspect (Figure 1).^1^ Tear of the transverse carpal ligament attachment at the trapezial ridge is rare and has previously only been described with associated carpal fractures or dislocations, and an avulsion fracture of the trapezial ridge.^2,3^ Injury to the transverse carpal ligament trapezial attachment without an associated trapezial ridge fracture has, to the authors’ best knowledge, not been previously described. We present two clinical cases, their diagnostic imaging findings and discuss the diagnostic imaging considerations and clinical implications of this injury.

## Case presentations

**Figure 2.**
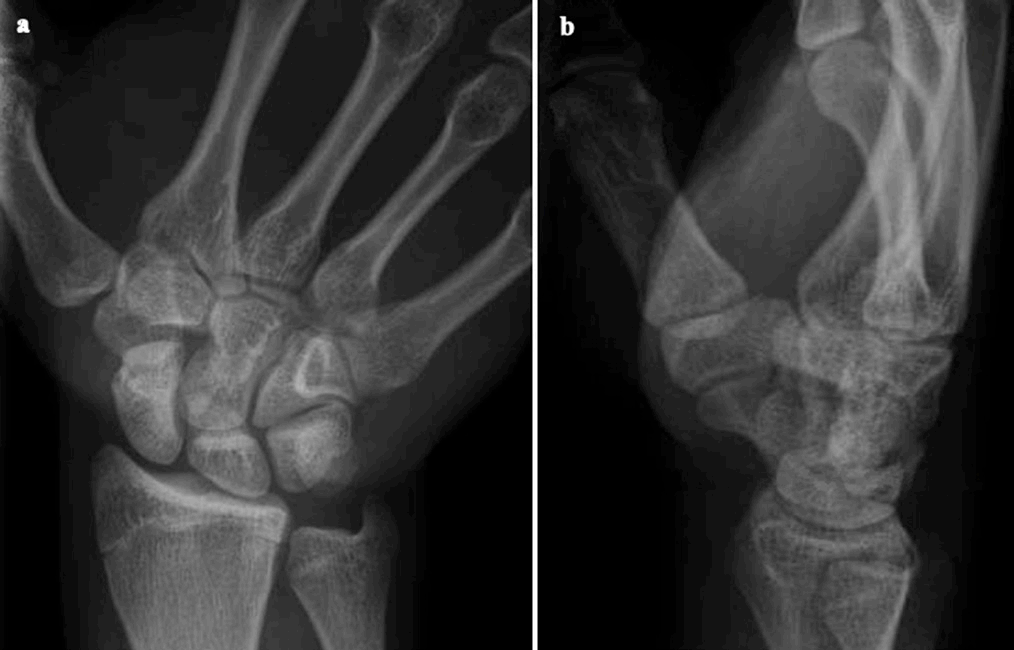
(**a**) Anteroposterior radiograph of the right wrist of the 16-year-old male patient. (**b**). Lateral radiograph of the right wrist of the 16-year-old male patient. No acute bone injury or malalignment were demonstrated on the radiographs.

**Figure 3.**
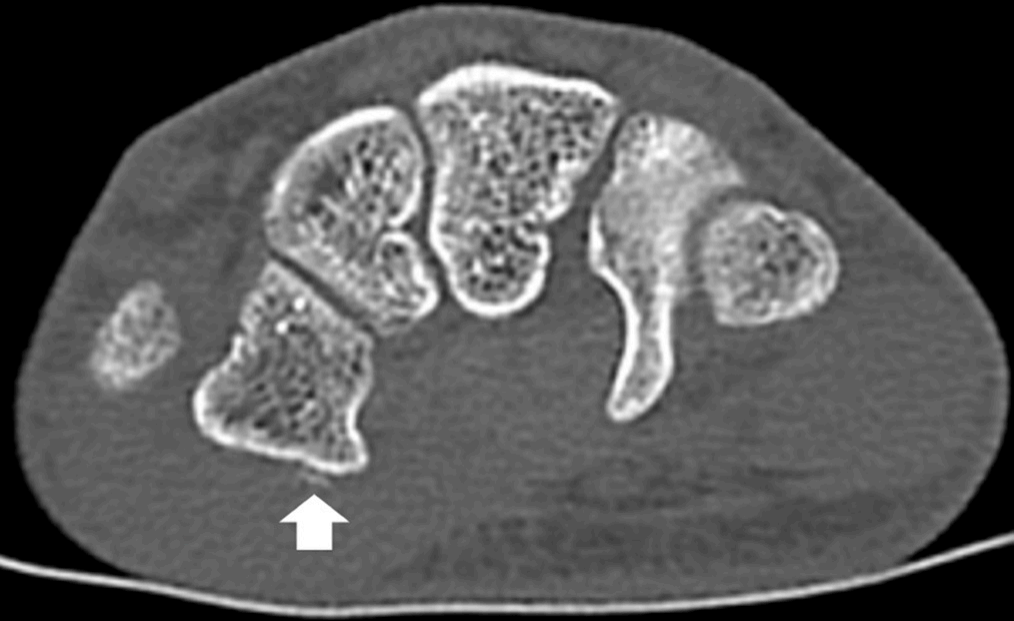
Axial computed tomography image of the right wrist of the 16-year-old male patient showing a 4-mm linear density (arrow) at the volar and lateral aspect of the trapezial ridge, which was interpreted as representing a minor degree of periosteal stripping or focal subperiosteal haemorrhage.

**Figure 4.**
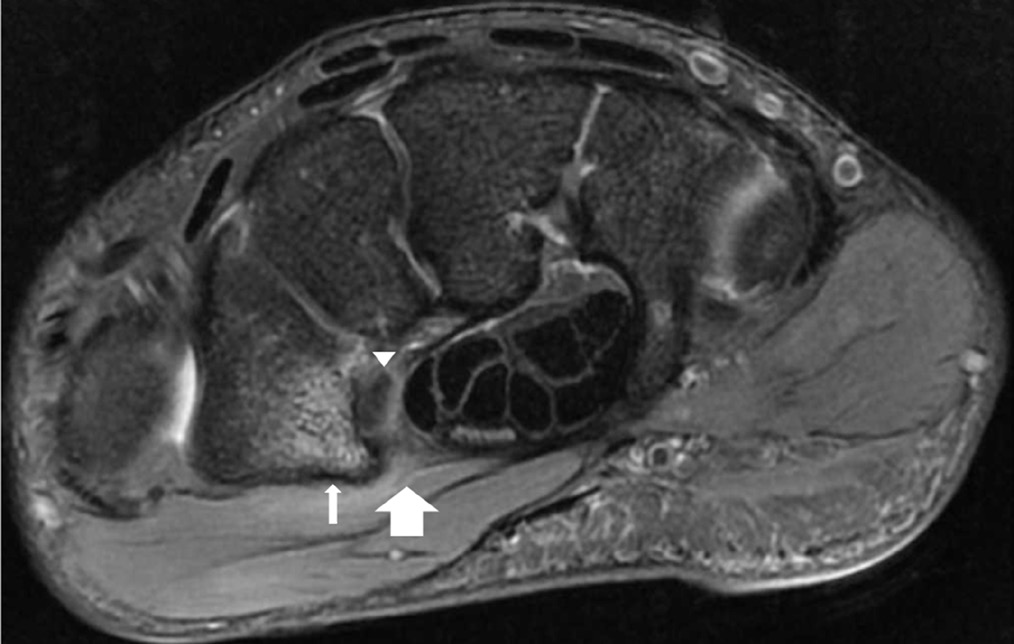
Axial proton density fat suppressed MRI image of the right wrist of the 16-year-old male patient demonstrating loss of the normal low signal intensity of the transverse carpal ligament attachment at the trapezial ridge representing a tear (large arrow). Bone marrow oedema within the volar aspect of the trapezium (small arrow) and mild oedematous change of the distal flexor carpi radialis tendon are also demonstrated (arrowhead).

A 16-year-old right-hand dominant Caucasian male patient, with no relevant significant past medical history, presented to the Minor Injuries Department ten weeks after a fall from a bicycle onto his outstretched right hand. The patient had been unable to continue cycling immediately after the injury but had gradually resumed cycling and sports in the interval time period until presenting to the Minor Injuries Department due to persistent symptoms. He had not required analgesia following the injury and his pain score was 3/10 on presentation. He was experiencing pain on flexion and extension of the wrist, and on clinical examination there was bony tenderness of the scaphoid tubercle and triquetrum but no wounds, erythema, deformity or neurovascular compromise. Radiographs of the wrist with additional scaphoid views were obtained, which did not demonstrate any acute bony injury or malalignment (Figure 2). A subsequent magnetic resonance imaging (MRI) study of the right wrist was performed three days later. Our local protocol includes a coronal T1, coronal short tau inversion recovery (STIR), axial proton density fat saturation (PD FS), sagittal PD FS and axial isometric gradient echo (GE) sequences. Initially, the transverse carpal ligament injury was not diagnosed on the MRI of the wrist. Lack of awareness of this injury pattern led to initial misinterpretation of marrow oedema-like signal at the volar aspect of the trapezium, including the ridge, as an isolated bone contusion or impaction fracture. Additional findings on the MRI study included mild increased oedema-like signal surrounding the distal flexor carpi radialis tendon in the trapezial groove, which was otherwise intact, bone marrow oedema-like signal on the lateral aspect of the scaphoid without a fracture, and mild increased fluid-like signal of the deep thenar eminence musculature. A subsequent non-enhanced computed tomography (CT) study, five weeks after the MRI scan demonstrated no fractures of the trapezial ridge or the scaphoid, but a minor 4-mm area mineralisation interpreted as periosteal stripping or subperiosteal haematoma at the volar aspect of the trapezium, lateral to the trapezial ridge and remote from the attachment of the transverse carpal ligament was identified (Figure 3). On further review of the MRI, the transverse carpal ligament was appreciated as being thickened and indistinct with increased intrinsic fluid-like signal on the axial PD FS and GE sequences at its attachment to the trapezial ridge (Figure 4). It was not in continuity at this site, in keeping with a full thickness tear of the attachment, with periosteal stripping of the adjacent trapezium.

The patient was initially managed with a Futura splint with a thumb spica for support at the Minor Injuries Department and advised to take over the counter analgesia as required and not to participate in sports until further outpatient clinical review. The patient was then reviewed in a Hand Trauma clinic one week after this presentation, where the Futura splint was replaced with a right scaphoid cast as per the suspected scaphoid injury pathway at our local institution. Five weeks after this, the patient’s symptoms had continued to improve and on removing the cast was mobilising his hand and wrist normally, he was discharged from the surgical clinic and referred for hand physiotherapy. The patient rehabilitated well and he was able to return to full activity.

**Figure 5.**
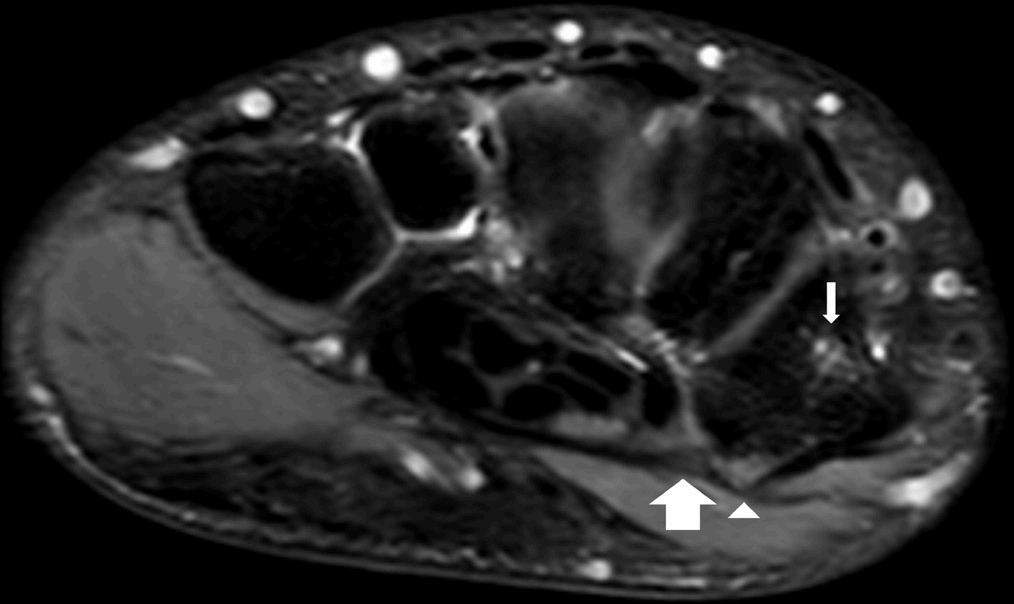
Axial proton density fat saturated MRI image of the left wrist of the 15-year-old male patient demonstrating increased signal intensity of the transverse carpal ligament attachment at the trapezial ridge representing an avulsion tear (large arrow) with an adjacent area of mild bone bruising at the trapezial ridge (arrow head) and the lateral aspect of the trapezium (small arrow).

The second case was a left-hand dominant 15-year-old Caucasian male patient, with no relevant significant previous medical history, who presented to the Emergency Department after falling off a bicycle onto his outstretched left hand. The patient’s left hand was bruised and swollen with a superficial wound over the dorsum of the left little finger at the level of the carpo-metacarpal joint. The anatomical snuff box was not tender, but there was tenderness of the scaphoid tubercle and on axial loading of the thumb, with reduced range of motion of the ring and little fingers. Radiographs of the left hand, including a scaphoid series, did not demonstrate a fracture. An MRI of the left wrist was obtained two and a half weeks later and the patient was diagnosed with a minor bone contusion at the lateral aspect of the scaphoid, and of the trapezium at the trapezial ridge, as well as a small displaced fracture of the dorsal aspect of the distal pole of the triquetrum. Following the first case, a second review of the MRI study was undertaken and a tear of the transverse carpal ligament at the attachment to the trapezial ridge was appreciated. A single key image for this ligament tear is presented in Figure 5.

The patient was given paracetamol, his wound was irrigated and dressed and he was discharged from the Emergency Department with a Futura splint. The patient was subsequently reviewed in the Hand Trauma clinic three weeks later, by which time his symptoms and range of motion of the wrist and hand had improved. He was referred to the hand physiotherapy services for further follow up.

## Discussion

We present the first reported acute tear of the transverse carpal ligament at the trapezial ridge. The strongest attachments of the transverse carpal ligament are at the hamate and trapezial ridge.^1^ Multiple articles describe avulsion fractures associated with the transverse carpal ligament attachment to the hamate and trapezium.^4,5^ One article has documented a tear of the transverse carpal ligament attachment at the hook of the hamate, without an accompanying fracture of the hamate, which was described as a focal linear area of high signal intensity on fluid-sensitive MRI sequences within the medial attachment of transverse carpal ligament. This was coined the ‘hook line’ sign or the ‘Salil-Zhang-Abhinav’ sign by the authors.^6^ Their description and published figures present comparable images to those cases presented in this article, a similar pattern on MRI of focal linear high fluid-like signal within the transverse carpal ligament at the lateral attachment to the trapezial ridge. We believe this has not been previously reported, and coin this injury pattern on MRI as the ‘trapezial ridge line’ sign.

An avulsion fracture of the trapezial ridge with associated transverse carpal ligament injury represents the main differential diagnosis when assessing for a possible transverse carpal ligament attachment tear.^2^ As demonstrated in our case, distinguishing between a trapezial ridge fracture and/or a transverse carpal ligament tear can be challenging. Lack of awareness of this pattern of ligament tear meant the diagnosis was only made retrospectively in both our presented cases. It is important to differentiate these entities as a fracture is likely to heal in 6 weeks whilst a ligament tear may take a longer timeframe to recuperate and may require a longer period of immobilisation in a cast.^4,7^ A tear without fracture is also occult on CT but detectable on MRI.

We postulate that this pattern of trapezial ligament injury, without an avulsion fracture, may be an injury of the adolescent patient group (as our two patient cases were 16 and 15 years of age at the time of injury) and is likely sustained following a low to medium energy injury, as in both our cases these injuries occurred after a fall onto the outstretched dominant-hand from a bicycle. Non-specific hand/wrist pain can be a major cause of morbidity, and it has been estimated this affects up to 10% of the general population.^8^ Ligamentous injuries such as the one presented here are likely underdiagnosed and therefore undertreated, and may contribute to this disease burden. Diagnosing ligamentous injuries also relies on MRI, as without a fracture these injuries are not detected on radiographs and CT, which are often utilised as first and second line imaging investigations at many medical institutions due to the comparatively higher cost and reduced availability of MRI services. The presented cases support the use of MRI as the second line diagnostic imaging modality of choice in wrist trauma patients with normal radiographs, as opposed to CT.^9,10^ Further research could be focused on longer term follow up to assess the risk factors and clinical outcomes of patients following a transverse carpal ligament tear, as well as research into further optimising an imaging pathway to aid in its diagnosis and exclusion of other differential injuries earlier and more cost-effectively.

## Learning points

1. We present two case examples and the first description of a transverse carpal ligament tear at its lateral attachment to the trapezial ridge without an associated avulsion fracture, and we introduce the MRI ‘trapezial ridge line’ sign.
2. Identifying this likely underdiagnosed tear has implications on the patient’s management, duration of immobilisation, including possible need for surgery, and stresses the value of MRI in evaluating the wrist in the acute trauma setting.
